# Barriers in the implementation of the Resuscitation Guidelines: European survey of defibrillation techniques

**DOI:** 10.1186/s13049-016-0219-2

**Published:** 2016-03-11

**Authors:** Paweł Krawczyk, Andrzej A. Kononowicz, Janusz Andres

**Affiliations:** Department of Anaesthesiology and Intensive Care, Jagiellonian University Medical College, Kopernika 17, 31-501 Krakow, Poland; Department of Bioinformatics and Telemedicine, Jagiellonian University Medical College, Lazarza 16, 31-530 Krakow, Poland

**Keywords:** Defibrillation, Technique, Adhesive pads, Manual paddles, Cardiopulmonary resuscitation, Advanced life support, Monitoring, Coupling medium

## Abstract

**Background:**

The European Resuscitation Council (ERC) Guidelines recommend providing chest compressions during defibrillator charging and using adhesive pads for defibrillation to increase the effectiveness of resuscitation. However, the most common defibrillation technique in each European country is unknown, as are the potential barriers in implementation of the guidelines. The aim of this study was to assess the techniques of defibrillation procedures performed by professional European healthcare providers and to estimate how frequently adhesive pads are used.

**Methods:**

We sent an online questionnaire to the ERC National Representatives that contained 12 questions regarding the techniques of defibrillation and monitoring heart rhythm during cardiac arrest. We also evaluated the frequency and indications of manual paddles use.

**Results:**

We collected questionnaires from 27 out of 33 invited ERC member countries. The response rate was 82 %. Seventeen (17/27; 63 %) declared the use of adhesive pads. The leading cause for not using adhesive pads was economic reason (9/17; 53 %). Some respondents declared resistance to using adhesive pads by healthcare providers or tradition connected with manual paddles use. We found three leading techniques of defibrillation with manual paddles: Charging paddles keeping them on the defibrillator during chest compressions being delivered (9/21; 43 %), Charging paddles keeping them on the patient chest during chest compressions being delivered (6/21; 29 %), Charging paddles on the patient chest without chest compressions (5/21; 24 %). Respondents from 11 countries declared the use of gel or electrode pastes during defibrillation with manual paddles.

**Discussion:**

This study collected preliminary data showing how defibrillation is performed in Europe. It revealed the recommeded techniques underuse and identyfied barriers in the Resuscitation Guidelines implementation. The survey should be open to a wider group of respondents. in each country in future.

**Conclusions:**

There are limitations and barriers in the implementation of the defibrillation technique guidelines. There are still countries where the use of adhesive pads is low due to economic and traditional reasons. There is a need for further efforts focused on guidelines implementation.

## Background

Performing defibrillation when a shockable rhythm has been identified is one of the key interventions of cardiac arrest with clearly proven benefits influencing patient survival [1]. The likelihood of a successful defibrillation attempt is lower not only when the procedure is done too late [2, 3], but also when there is a delay between stopping chest compressions and shock delivery [4–6]. A pause longer than 5–10 s may influence defibrillation effectiveness [7]. The European Resuscitation Council (ERC) Guidelines [1] recommend providing chest compressions during defibrillator charging to eliminate unnecessary breaks in chest compressions and to decrease the time between the stopping of CPR and shock delivery. The guidelines also recommend the use of adhesive pads for defibrillation. This has the potential to make the procedure quicker, safer and more effective than with manual paddles [8–10]. The ERC Guidelines for Resuscitation 2015 recognize that defibrillator paddles are used in some settings [1]. The use of paddles is still common in many European countries [11–13], however, it is not known what the most common defibrillation technique is in each country or what the potential barriers in implementation of the guidelines are.

This paper provides information regarding the technique of defibrillation procedures carried out by professional European healthcare providers in both pre- and in-hospital cardiac arrests. It also indicates the barriers in implementation of the guidelines regarding the use of adhesive pads.

## Methods

In January 2016, we sent an e-mail with invitation to participate in the online survey [14] to all ERC National Representatives – one per each member country. The questionnaire contained 12 questions regarding the technique of defibrillation and how heart rhythm is monitored during cardiac arrest. Additional questions were asked in regard to manual paddles use (indications, coupling medium usage, technique of manual defibrillation). The study questionnaire was created based on results and conclusions of the previous studies [11–13] and after consulting it with the then ERC Director of Science and Research. In case of no response within one week we sent up to 3 reminders. When still not successful we sent the invitation to the missing national resuscitation councils’ secretariat or second contact person if available.

## Results

**Fig. 1:**
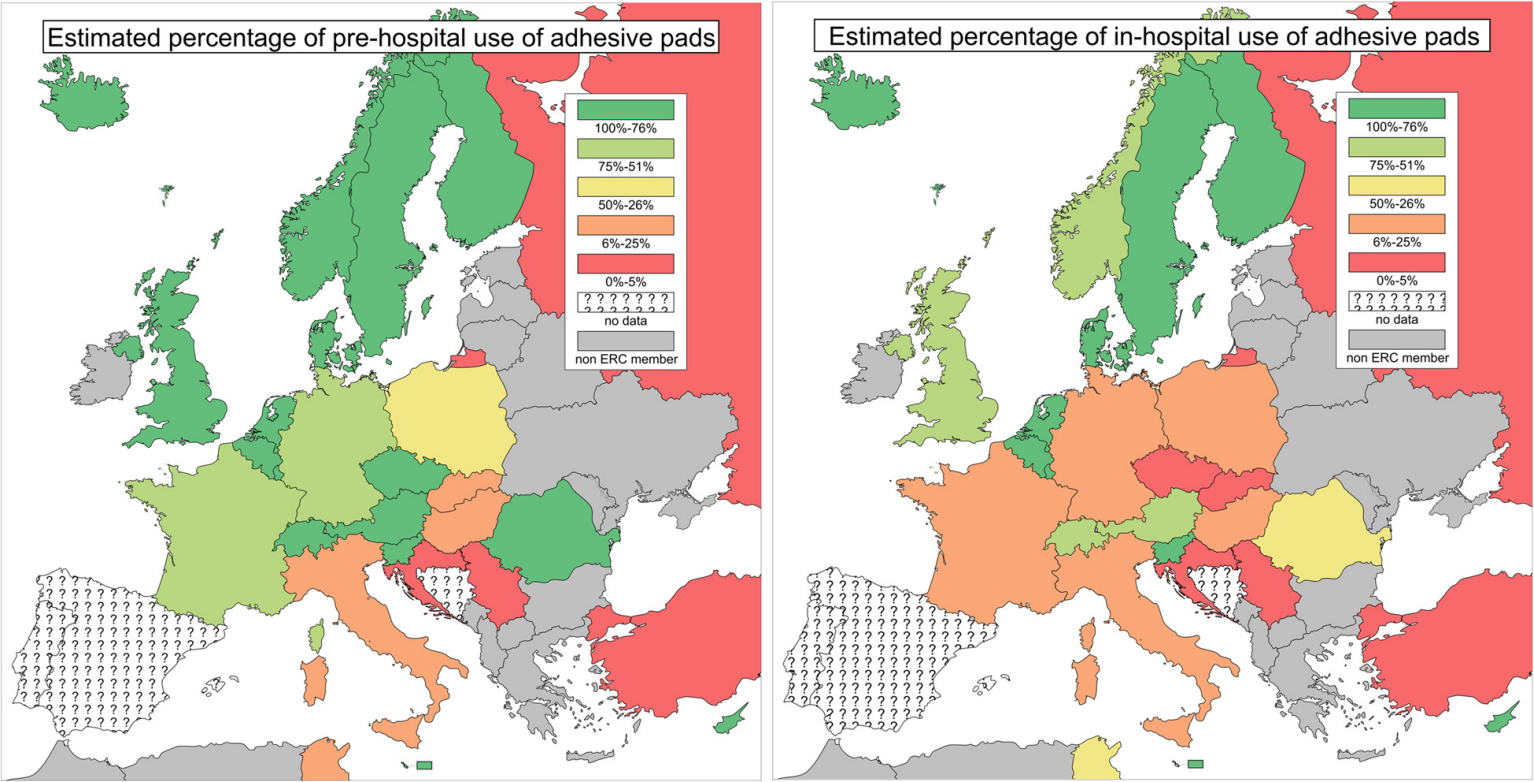
Responses on technique of defibrillation used in pre-hospital and in-hospital areas

We collected questionnaires from 27 out of 33 ERC member countries: Austria, Belgium, Croatia, Cyprus, Czech Republic, Denmark, Finland, France, Germany, Hungary, Iceland, Italy, Luxembourg, Malta, Norway, the Netherlands, Poland, Romania, Russian Federation, Serbia, Slovakia, Slovenia, Sweden, Switzerland, Turkey, Tunisia and the United Kingdom. The following countries did not respond: Bosnia and Herzegovina, Egypt, Portugal, Spain, Sudan and United Arabic Emirates (Fig. 1). The response rate was 82 %. The respondents were physicians (24/27; 89 %), two nurses and a paramedic. Most of the respondents worked in a hospital only (13/27; 48 %), both in- and out-of-hospital worked 8/27 (30 %) and 6/27 (22 %) worked outside of the hospital only.

Sixty-three percent of the respondents (17/27) declared using adhesive pads as the defibrillation technique. In the opinion of the respondents adhesive pads are used more often in pre-hospital cardiac arrest than in-hospital (the frequency of use 76–100 % was estimated for pre-hospital 16/27 (59 %) vs. in-hospital 10/27 (37 %)). Figure 1 presents responses regarding the technique of defibrillation used pre-hospital and in-hospital. There were 10/27 (37 %) countries declaring use of adhesive pads in both pre- and in-hospital environment in a rate of 76–100 %. The leading cause indicated by respondents for not using adhesive pads in their country was for economic reasons 9/14 (64 %). Three (3/14; 21 %) respondents declared a resistance to the use of adhesive pads by healthcare providers or a tradition connected with using manual paddles. In cases of using manual paddles, 7/23 (30 %) respondents declared use of gel and 4/23 (17 %) electrode pastes as a coupling medium and 48 % (11/23) used gel pads. In the free text comments, some respondents declared a problem with the availability of gel pads in their country.

We found three leading techniques of manual defibrillation with paddles (21 responses):

A. Charging paddles keeping them on the defibrillator during chest compressions being delivered (9/21; 43 %)
B. Charging paddles keeping them on the patient’s chest during chest compressions being delivered (6/21; 29 %)
C. Charging paddles on the patient chest without chest compressions (5/21; 24 %)

As a free text comment, one respondent stated that “there was no uniform practice”.

The respondents judged the benefits of the chosen defibrillation technique with manual paddles selecting high chest compression quality during charging paddles in 7/17 (41 %), a short time from chest compression cessation to shock delivery in 5/17 (29 %), and safety of the rescuer in 2/17 (12 %) of responses.

Cardiac arrest rhythm was initially assessed using adhesive pads by 15/27 (56 %) of respondents, 6/27 (22 %) declared use of the quick-look technique for that purpose and 5/27 (19 %) preferred 3-lead ECG for initial monitoring of cardiac arrest. As a free text comment, one respondent stated that “there was no standard practice, depending on people involved and equipment available”.

The detailed results of this part of the survey are available in Table 1.

**Table 1:** Results of the defibrillation techniques survey 2016. Questions 1-9

#	Question	Response	n	%
1.	How do you usually perform defibrillation in your department/working place:			*N* = 27
		adhesive pads	17	63 %
		manual paddles	10	37 %
2.	What do you estimate is the percentage of using adhesive pads in your country: Pre-hospital			*N* = 27
		76–100 %	16	59 %
		51–75 %	2	7 %
		26–50 %	1	4 %
		6–25 %	4	15 %
		0–5 %	4	15 %
3.	What do you estimate is the percentage of using adhesive pads in your country: In-hospital			*N* = 27
		76–100 %	10	37 %
		51–75 %	4	15 %
		26–50 %	2	7 %
		6–25 %	5	19 %
		0–5 %	6	22 %
4.	If adhesive pads use in your country is lower than 76–100 % please specify the main reason for that			*N* = 14
		Economic reasons	9	64 %
		Resistance to use adhesive pads by healthcare providers	3	21 %
		Other, please specify in box next to the question	2	14 %
5.	What coupling medium do you use in order to facilitate manual defibrillation with paddles in your country?			*N* = 23
		Electrode pastes	4	17 %
		Gel	7	30 %
		Gel pads	11	48 %
		Other	1	4 %
6.	If you are using manual paddles what technique of defibrillation is preferred in your country			*N* = 21
		Charging paddles keeping them on the defibrillator during chest compressions being delivered	9	43 %
		Charging paddles keeping them on the patient chest during chest compressions being delivered	6	29 %
		Charging paddles on the patient chest without chest compressions	5	24 %
		Other technique (please describe):	1	5 %
7.	What major benefit of chosen manual defibrillation technique with paddles you find useful			*N* = 17
		High chest compressions quality delivered during charging paddles	7	41 %
		Safety - no risk for rescuer	2	12 %
		Short time from chest compressions cessation to shock delivery	5	29 %
		Other (please specify)	3	18 %
8.	What major drawback of other defibrillation techniques with paddles make them useless for you			*N* = 20
		Delay from chest compressions cessation to shock delivery	8	40 %
		Poor chest compressions quality delivered during charging paddles	4	20 %
		Risk for rescuer	7	35 %
		Other (please specify)	1	5 %
9.	If healthcare providers in your country use the quick-look with manual paddles technique for the FIRST assessment in cardiac arrest victim, what would be the next step when confirming shockable rhythm			*N* = 16
		Charge the defibrillator with paddles ON the patient chest WITH ongoing chest compressions	2	13 %
		Charge the defibrillator with paddles ON the patient chest WITHOUT chest compressions	5	31 %
		Remove paddles from the patient chest and resume/start chest compressions, than deliver shock after charging defibrillator	5	31 %
		Other technique, please describe in box on the right hand side	4	25 %

When the quick-look technique was used and shockable rhythm was detected, the next suggested step from 2/16 (13 %) of respondents was charging the defibrillator with the paddles on the patient’s chest with ongoing chest compressions, however 5/16 (31 %) of respondents declared charging the defibrillator with paddles on the patient’s chest without chest compressions. Five out of 16 (31 %) of respondents suggested removing the paddles from the patient’s chest and resuming/starting chest compressions, and then delivering a shock after charging the defibrillator. The technique for further monitoring of cardiac arrest rhythm and the modification of the monitoring in case of low ECG signal quality is shown in Table 2.

**Table 2:** The way of monitoring cardiac arrest rhythm. Results of the defibrillation techniques survey 2016. Questions 10–12

#		Adhesive pads	“Quick look” with paddles	3-lead ECG	Other	No change in monitoring technique
10.	Cardiac arrest rhythm initial assessment	56 % (15/27)	22 % (6/27)	19 % (5/27)	4 % (1/27)	N/A
11.	Best signal quality for monitoring during cardiac arrest	37 % (10/27)	4 % (1/27)	56 % (15/27)	4 % (1/27)	N/A
12.	Change in monitoring in case of low ECG signal quality	26 % (7/27)	11 % (3/27)	37 % (10/27)	7 % (2/27)	19 % (5/27)

(N/A – not applicable)

In free text comments regarding the technique of monitoring cardiac rhythm, there were suggestions that adhesive pads provide a good ECG signal, that changing ECG leads may be a solution for poor ECG signal quality and that more modern equipment will result in a better quality of ECG.

## Discussion

The Resuscitation Guidelines recommend minimizing unnecessary breaks in chest compression delivery [1]. When defibrillation is attempted, the time from cessation of chest compressions to shock delivery should not exceed 5–10 s, which is possible if chest compressions are performed while charging the defibrillator. Despite the fact that guidelines recommend using adhesive pads, the survey results revealed that the use of manual paddles is still common in Europe. There were just 10/27 (37 %) countries declaring use of adhesive pads in both pre- and in-hospital environment in a rate of 76–100 %. The use of adhesive pads was declared by 63 % of the respondents. There are discrepancies in the use of adhesive pads between the countries and the location of the healthcare service (pre- and in-hospital areas). In countries with low adhesive pads usage, we see signs of growing use of adhesive pads in the pre-hospital area, which may be associated with awareness of the guidelines in this group of healthcare providers which changes the former defibrillation practice [11–13]. In the recently published study from Hungary only 6,5 % of the interviewed senior consultants of the intensive care units and emergency departments from audited 56 hospitals declared the use of adhesive pads routinely at the time of the survey [15].

The 2015 Resuscitation Guidelines recognize a possibility of providing defibrillation with manual paddles in case of lack of adhesive pads [1], however, there is limited number of evidence how frequent it happens and what is the technique of defibrillation performed with manual paddles since the Guidelines do not provide any information regarding this topic. Based on previous reports on this topic we identified the most common techniques of performing defibrillation. The questionnaire contained a field for any other technique, however, no respondent suggested a different approach. In our study three different techniques of manual paddles use were reported. Two of them include charging the defibrillator during chest compression delivery. The difference between them is the location of the paddles – either on the patient’s chest (29 %) or on the defibrillator (43 %). Currently, there is no evidence to determine which technique is better in terms of chest compression quality, safety of the rescuer and pre- and post-shock pauses in chest compressions.

Five of the respondents declared they did not perform chest compressions during charging the defibrillator, which, despite the short time it takes for charging of the modern defibrillator, still generates long pauses in chest compression delivery. The survey was performed 3 months after delivery of the 2015 Guidelines, however, the 2005 technique was still reported as being used, which may reflect current practice in some European countries. There are also differences in performing defibrillation with adhesive pads (charging the defibrillator towards the end of every 2 min cycle of CPR). This is also recognized in the 2015 guidelines by the ALS writing group, however, the benefit from this intervention is unknown [1, 16, 17].

Interestingly, an economic reason for not using adhesive pads, even though high 9/14 (64 %), was not the only reason indicated by respondents. Resistance to using adhesive pads and the tradition of using manual paddles are still important barriers in guideline implementation. The Hungarian study also revealed that the major obstacle for adhesive pads use which were the perceived cost-efficiency concerns declared in 60 % of responds, however, the majority of clinicians (92 %) were aware of the benefits of adhesive pads use [15].

Many of the respondents (47 %) using the manual paddles still use gel or electrode pastes as a coupling medium, despite the fact it is not recommended since 2005 Guidelines release [18]. The reason for that may be low availability of gel pads is some European countries indicated by the respondents.

The use of adhesive pads is the leading technique for initial cardiac arrest rhythm assessment (56 % of respondents), however, the quick-look technique appears to be used at a similar level (22 %) with 19 % of respondents declaring 3-lead ECG use for initial monitoring. The choice of the different approach to initial cardiac rhythm assessment may influence the outcome of resuscitation. One of the key changes in ERC ALS Guidelines since 2010 is keeping the focus on the use of adhesive pads and a defibrillation strategy to minimize the preshock pause [1]. The use of 3-lead ECG monitoring for initial rhythm assessment is definitely inferior to either adhesive pads or “quick look” with paddles [9], however, five of the respondents who declared adhesive pads use chose 3-lead ECG monitoring for that purpose.

There are also different approaches to the technique of defibrillation attempts when shockable rhythm is present during the quick-look assessment technique. Further studies are needed to indicate the optimal approach.

When a low quality signal was detected, 10/27 (37 %) of the survey respondents changed the method of monitoring to 3-lead ECG, 7/27 (26 %) looked for better signal quality with adhesive pads use and 5/27 (19 %) of respondents did not change the method of monitoring. The quality of the ECG signal is vital during cardiac arrest management and may influence therapeutic decisions. Currently, there are no human studies known to the authors of this paper assessing this issue in terms of cardiac arrest management.

### Study limitations

The study is limited by the small number of respondents: only those ERC National Representatives who decided to respond to the on-line questionnaire. We did not receive responses from all European countries which may generate a bias. Some respondents, however, representing their country, have found difficulties in indicating the exact percentage of adhesive pads use in their countries which hinders drawing clear conclusions. On the other hand it was the only way to collect preliminary data showing how defibrillation is performed in Europe. There were suggestions from the survey participants that the study should be open to a wider group of respondents in each country.

## Conclusions

Based on the observations as presented above, we conclude that there are limitations and barriers in implementation of the defibrillation technique guidelines. There are still countries where the use of adhesive pads is low due to economic and traditional reasons. There is a need for further efforts focused on guidelines implementation in terms of the use of adhesive pads and a defibrillation strategy to minimize the preshock pause.

## References

[1] Soar J, Nolan JP, Böttiger BW, Perkins GD, Lott C, Carli P (2015). European Resuscitation Guidelines for Resuscitation 2015. Section 3. Adult advanced life support. Resuscitation..

[2] Larsen MP, Eisenberg MS, Cummins RO, Hallstrom AP (1993). Predicting survival from out-of-hospital cardiac arrest: a graphic model. Ann Emerg Med.

[3] Valenzuela TD, Roe DJ, Cretin S, Spaite DW, Larsen MP (1997). Estimating effectiveness of cardiac arrest interventions: a logistic regression survival model. Circulation.

[4] Edelson DP, Abella BS, Kramer-Johansen J, Wik L, Myklebust H, Barry AM (2006). Effects of compression depth and pre-shock pauses predict defibrillation failure during cardiac arrest. Resuscitation.

[5] Eftestøl T, Sunde K, Steen PA (2002). Effects of interrupting precordial compressions on the calculated probability of defibrillation success during out-of-hospital cardiac arrest. Circulation.

[6] Gundersen K, Kvaløy JT, Kramer-Johansen J, Steen PA, Eftestøl T (2009). Development of the probability of return of spontaneous circulation in intervals without chest compressions during out-of-hospital cardiac arrest: an observational study. BMC Med..

[7] Deakin CD, Nolan JP, Sunde K, Koster RW (2010). European Resuscitation Council Guidelines for Resuscitation 2010 Section 3. Electrical therapies: automated external defibrillators, defibrillation, cardioversion and pacing. Resuscitation.

[8] Perkins GD, Davies RP, Soar J, Thickett DR (2007). The impact of manual defibrillation technique on no-flow time during simulated cardiopulmonary resuscitation. Resuscitation.

[9] Perkins GD, Roberts C, Gao F (2002). Delays in defibrillation: influence of different monitoring techniques. Br J Anaesth.

[10] Stults KR, Brown DD, Cooley F, Kerber RE (1987). Self-adhesive monitor/defibrillation pads improve prehospital defibrillation success. Ann Emerg Med.

[11] Krawczyk P, Kononowicz AA, Andres J (2012). Manual defibrillation technique – A pilot survey of European performance – poster presentation abstract. Resuscitation..

[12] Cebula G, Koszowski P, Krawczyk P, Kononowicz AA, Odrzywołek R, Andres J (2011). Manual defibrillation according to the 2010 European Resuscitation Council (ERC) Guidelines – is there a consensus? Report from 6th International Emergency Medicine Championship. Resuscitation..

[13] Cebula G, Krawczyk P, Kononowicz AA, Koszowski M, Odrzywołek R, Andres J (2012). Manual defibrillation using paddles ‐ Which is the best technique?. Resuscitation..

[14] The archived Defibrillation Technique Survey 2016 questionnaire: http://bioinformatics.cm-uj.krakow.pl/limesurvey/index.php?sid=35971&lang=en Accessed on 9th February 2016.

[15] Dioszeghy C, Molnar N (2014). Current practice and perspective of hands-free defibrillation in Hungary – Investigating the obstacles of implementation. Interv Med Appl Sci.

[16] Edelson DP, Robertson-Dick BJ, Yuen TC, Eilevstjønn J, Walsh D, Bareis CJ (2010). Safety and efficacy of defibrillator charging during ongoing chest compressions: a multi-center study. Resuscitation.

[17] Hansen LK, Mohammed A, Pedersen M, Folkestad L, Brodersen J, Hey T et al. The Stop-Only-While-Shocking algorithm reduces hands-off time by 17 % during cardiopulmonary resuscitation - a simulation study. Eur J Emerg Med 2015. [Epub ahead of print] 10.1097/MEJ.0000000000000282 25951368

[18] Nolan JP, Deakin CD, Soar J, Bottiger BW, Smith G (2005). European Resuscitation Council Guidelines for Resuscitation 2005 Section 4. Adult advanced life support. Resuscitation.

